# Tissue Adhesive, Biocompatible, Antioxidant, and Antibacterial Hydrogels Based on Tannic Acid and Fungal-Derived Carboxymethyl Chitosan for Wound-Dressing Applications

**DOI:** 10.3390/gels9050354

**Published:** 2023-04-22

**Authors:** Kummara Madhusudana Rao, Uluvangada Thammaiah Uthappa, Hyeon Jin Kim, Sung Soo Han

**Affiliations:** 1 School of Chemical Engineering, Yeungnam University, 280 Daehak-Ro, Gyeongsan 38541, Gyeongbuk, Republic of Korea; sanjuuthappa@gmail.com (U.T.U.); hyeonjin3438@naver.com (H.J.K.); 2 Research Institute of Cell Culture, Yeungnam University, 280 Daehak-Ro, Gyeongsan 38541, Gyeongbuk, Republic of Korea

**Keywords:** tissue adhesive, biocompatible, antibacterial, antioxidant, hydrogel, wound dressing

## Abstract

This study aimed to develop hydrogels for tissue adhesion that are biocompatible, antioxidant, and antibacterial. We achieved this by using tannic acid (TA) and fungal-derived carboxymethyl chitosan (FCMCS) incorporated in a polyacrylamide (PAM) network using free-radical polymerization. The concentration of TA greatly influenced the physicochemical and biological properties of the hydrogels. Scanning electron microscopy showed that the nanoporous structure of the FCMCS hydrogel was retained with the addition of TA, resulting in a nanoporous surface structure. Equilibrium-swelling experiments revealed that increasing the concentration of TA significantly improved water uptake capacity. Antioxidant radical-scavenging assays and porcine skin adhesion tests confirmed the excellent adhesive properties of the hydrogels, with adhesion strengths of up to 39.8 ± 1.2 kPa for 1.0TA-FCMCS due to the presence of abundant phenolic groups on TA. The hydrogels were also found to be biocompatible with skin fibroblast cells. Furthermore, the presence of TA significantly enhanced the antibacterial properties of the hydrogels against both Gram-positive (*Staphylococcus aureus*) and Gram-negative (*Escherichia coli*) bacteria. Therefore, the developed drug-free antibacterial and tissue-adhesive hydrogels can potentially be used as wound dressings for infected wounds.

## 1. Introduction

Hydrogels are water-absorbing polymers that can absorb and retain large amounts of water, making them useful for wound-dressing applications [1]. They are soft and flexible, making them comfortable to wear, and they can be designed to release drugs or other therapeutic agents to aid in wound healing [2]. So far, various types of hydrogel dressings have been developed in different forms, such as films, sheets, gels, foams, and nanoparticles [3]. They can be designed to have different mechanical properties, such as stiffness and elasticity, in order to match specific wound types and stages of healing [3,4]. Furthermore, the use of hydrogels is of particular interest with respect to wound-dressing because they can provide a moist wound-healing environment, which is optimal for wound healing. They can also provide a barrier against infection and help to reduce pain and inflammation. Some hydrogels can be designed to promote the growth of new tissue and blood vessels, which can help accelerate wound healing. So far, antibiotic-drug-loaded hydrogels have been studied as a potential treatment option for infected wounds. These hydrogels are designed to release antibiotics over a sustained period, providing a high concentration of the drug directly to the wound site. However, their use must be carefully balanced against the risk of allergic reactions and antibiotic resistance [5,6]. It is important to carefully select the appropriate antibiotic, consider the risk of antibiotic resistance, and monitor the patient for any adverse effects. In this regard, drug-free antibacterial hydrogels have more benefits for wound-dressing applications.

There are several advantages of using natural polymers instead of synthetic polymers for wound-dressing applications [7]. Some of these advantages include biocompatibility, biodegradability, non-toxicity, natural healing properties, and sustainability. Among the various types of natural polymers, chitosan (CS) is a natural polymer derived from chitin, which is a major component of the exoskeletons of crustaceans, insects, and fungi [8]. Carboxymethyl chitosan (CMCS) is a water-soluble derivative of CS that has been modified by carboxymethylation, resulting in a more hydrophilic and water-soluble material [9]. CMCS hydrogels can be synthesized by crosslinking CMC with a crosslinking agent, such as glutaraldehyde or genipin [10]. The resulting hydrogel has high water content and can absorb and retain large amounts of fluid, providing a moist wound-healing environment. One of the advantages of CMCS hydrogels is their antimicrobial activity. Studies have shown that CMCS hydrogels can inhibit the growth of various bacteria and fungi, including *Staphylococcus aureus* and *Candida albicans*, which are common wound pathogens [11]. In addition to their antimicrobial properties, CMCS hydrogels can also promote wound healing. CMCS has been shown to enhance the proliferation and migration of fibroblasts, which are essential for the formation of new tissue. It can also stimulate the production of collagen and other extracellular matrices (ECM) proteins, which provide structural support to a wound. Recently, fungal-derived carboxymethyl chitosan (FCMCS) hydrogels have been studied in relation to their potential application as wound dressings [12,13,14,15]. FCMCS hydrogels have the additional advantage of being derived from a sustainable and renewable source. Fungi are fast-growing and can be easily cultured in large quantities, making FCMCS an eco-friendlier alternative to CS derived from crustaceans. Overall, FCMCS hydrogels constitute a promising technology for wound dressing, which can potentially improve wound-healing outcomes and reduce the risk of infection [12,13,14,15]. 

Adhesive hydrogels are hydrophilic materials designed to adhere to surfaces such as skin or tissue without causing discomfort or damage. These hydrogels have adhesive and cohesive properties, enabling them to stick to a surface while maintaining structural integrity. Adhesive hydrogels have widespread applications in biomedical fields, such as wound healing, drug delivery, and tissue engineering. They provide a moist wound-healing environment and can directly deliver therapeutic agents to a wound or tissue defect site. Designing adhesive hydrogels presents a challenge with respect to balancing strong adhesion and gentle removal. This balance is necessary to keep the hydrogel in place without causing pain or damage during removal [16,17]. Researchers have developed various approaches to address this challenge, including incorporating adhesion-promoting molecules, designing hydrogels with hierarchical structures, or using reversible adhesion strategies. Tannic acid (TA) is a natural polyphenolic compound found in several plants, including tea, coffee, grapes, and oak bark, that has been shown to adhere to tissues in TA-based hydrogels [18,19,20]. These hydrogels are formed by crosslinking TA with a polymer matrix such as polyvinyl alcohol or CS, resulting in high water content that allows for the absorption and retention of large amounts of fluid, which promotes moist wound healing. TA hydrogels possess antibacterial and anti-inflammatory properties, allowing them to inhibit the growth of various bacteria, including Staphylococcus aureus, and scavenge free radicals while reducing the release of inflammatory cytokines. Additionally, TA can enhance the proliferation and migration of fibroblasts, which are essential for forming new tissue, while stimulating collagen production to provide structural support to a wound [21]. So far, various types of TA-based hydrogels loaded with silver nanoparticles and other antibiotic drugs have been developed for wound-dressing applications [18,19,20]. One issue with silver nanoparticles is their potential toxicity to human cells, particularly at high concentrations or after prolonged exposure. The use of silver nanoparticles in wound dressings has raised concerns about the potential for these particles to enter the bloodstream and accumulate in the body, which could harm a patient. Another potential problem with drug-loaded TA-based hydrogels is the risk of adverse effects posed by the drug itself. Depending on the type and dose of drug used, patients may experience side effects such as allergic reactions or systemic toxicity. Additionally, the efficacy of the drug may be limited by its ability to penetrate the wound bed and reach the target tissue. Thus, the use of drug-free approaches via the employment of antibacterial polymers based on CMCS offer more advantages with respect to improving biocompatibility and enhancing wound healing [12,13,14,15]. 

By considering the potential benefits of FCMCS and TA for wound healing, in this study, we developed FCMCS- and TA-based drug-free hydrogels with antibacterial and tissue adhesive properties for wound-dressing applications. We expect that the combination of TA and FCMCS can result in a synergistic effect, thereby enhancing the properties of the hydrogel. TA can act as a crosslinking agent, promoting the formation of a stable hydrogel network. FCMCS can improve the water retention capacity of a hydrogel as well as its mechanical strength and elasticity. Overall, adhesive hydrogels constitute a promising technology that can potentially improve biomedical applications, especially in wound healing and tissue engineering.

## 2. Results and Discussion

### 2.1. Preparation of TA-FCMCS Hydrogels and Its Characterization

**Scheme 1:**
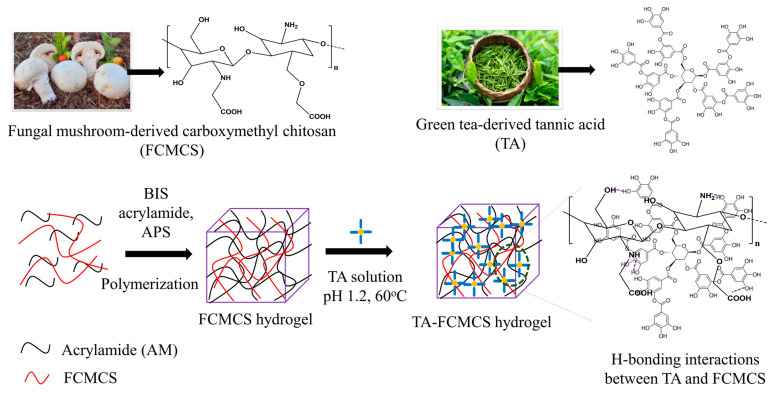
Schematic representation of formation of TA-FCMCS hydrogel.

A simple procedure has been employed for the preparation of TA-FCMCS hydrogels for wound-dressing applications. In the first step, FCMCS hydrogels were prepared using an acrylamide (AM) monomer, N,N′-methylene-bis(acrylamide) (BIS) as a crosslinker in the presence of ammonium persulfate (APS) as an initiator. The as-prepared FCMCS hydrogels were soaked in TA solutions at 60 °C under acidic conditions (pH 1.2) for 12 h. During this process, the functional groups that existed in the FCMCS (-COOH, -NH_2_, -OH) and PAM networks (-CO-NH_2_) easily interacted with the phenolic -OH groups of TA via H-bonding interactions, thereby improving their physicochemical and biological properties (Scheme 1).

**Figure 1:**
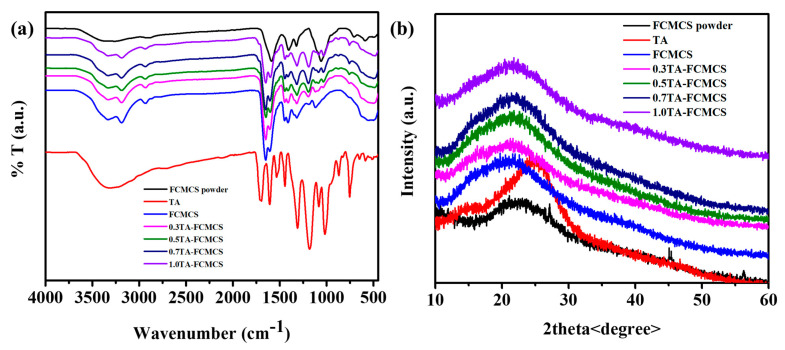
(**a**) FTIR spectra and (**b**) XRD patterns of hydrogels.

The formation of the FCMCS and TA-FCMCS hydrogels was characterized using FTIR spectroscopy. The FTIR spectra of the pure FCMCS powder displayed distinctive peaks at 3408 cm^−1^, which were attributed to –OH and NH_2_ stretching vibrations, and at 1588 and 1408 cm^−1^, which were ascribed to COO^−^ asymmetric and symmetric stretching vibrations. Additionally, peaks at 1064 and 1411 cm^−1^ were observed, representing C-O and C-OH functional groups, respectively. In the FTIR spectrum of the FCMCS hydrogel, peaks at 1650 cm^−1^ and 1605 cm^−1^ were assigned to C=O stretching and N-H deformations, while a peak at 1412 cm^−1^ represented C-N stretching vibrations. The TA sample exhibited a prominent peak at 1718 cm^−1^, corresponding to catechol stretching vibrations. Other peaks observed at 1613, 1533, and 1448 cm^−1^ were attributed to benzyl stretching vibrations. In the TA-FCMCS hydrogel, the peak intensity of catechol’s stretching vibration was decreased, and similar peaks belonging to PAM and FCMCS were observed. Increasing the concentration of TA in the FCMCS hydrogels caused all the peaks to shift to lower stretching frequencies, suggesting H-bonding interactions between TA (-OH) and the functional groups present in the FCMCS and PAM network structures [22,23,24]. The XRD patterns of the hydrogels are displayed in Figure 1b. The XRD patterns of the FCMCS powder and TA samples showed peaks at 2theta of 22° and 25°, indicating their semi-crystalline nature. The XRD patterns of the FCMCS hydrogel showed a peak at 2theta of 20°, suggesting its amorphous nature. The addition of TA also showed a similar amorphous peak, indicating molecularly dispersed interactions of TA within the hydrogel structure [24].

**Figure 2:**
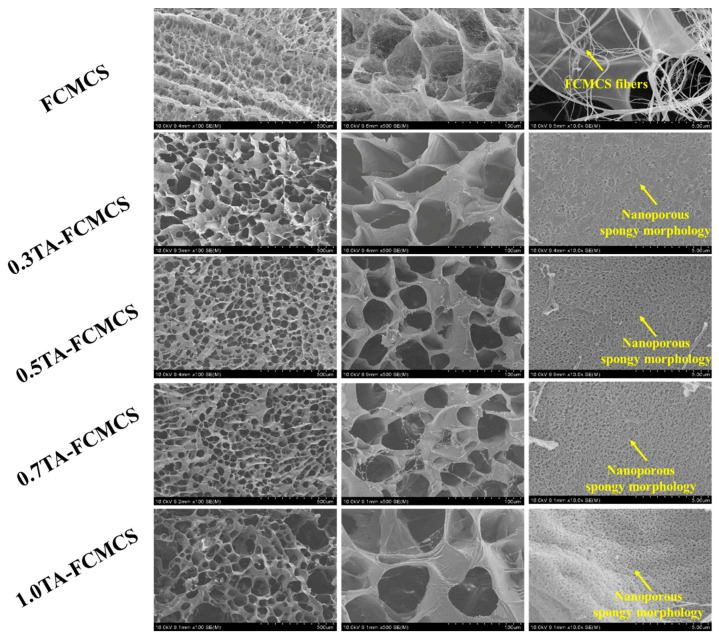
SEM images of hydrogels.

SEM images are a valuable tool for analyzing the structures and properties of hydrogel materials. Figure 2 shows SEM images of the TA-FCMCS hydrogels with varying amounts of TA. The resulting images reveal that the lyophilized FCMCS hydrogel exhibited a three-dimensional network structure with interconnected regular pores and FCMCS fiber structures with smooth surfaces [13]. In contrast, the lyophilized TA-FCMCS hydrogel had a similar porous structure with a nanofibrous surface topology. It is worth noting that the fibrous structure of FCMCS was destroyed in the TA-FCMCS hydrogel due to the formation of hydrogen-bonding interactions between TA and FCMCS during the soaking of the hydrogel in the TA solutions. Interestingly, a nanofibrous surface topology was observed for all the TA-FCMCS hydrogel samples, with the nanofibrous topology becoming more apparent with increasing amounts of TA due to the hydrogen-bonding interactions between TA and FCMCS [20]. The appearance of clear pores and a nanofibrous surface topology is beneficial for retaining wound moisture for longer periods and removing wound extrudate after the removal of the dressing at the wound site [25]. Furthermore, these structures would be beneficial for cell growth for new ECM modeling.

### 2.2. Swelling Study

**Figure 3:**
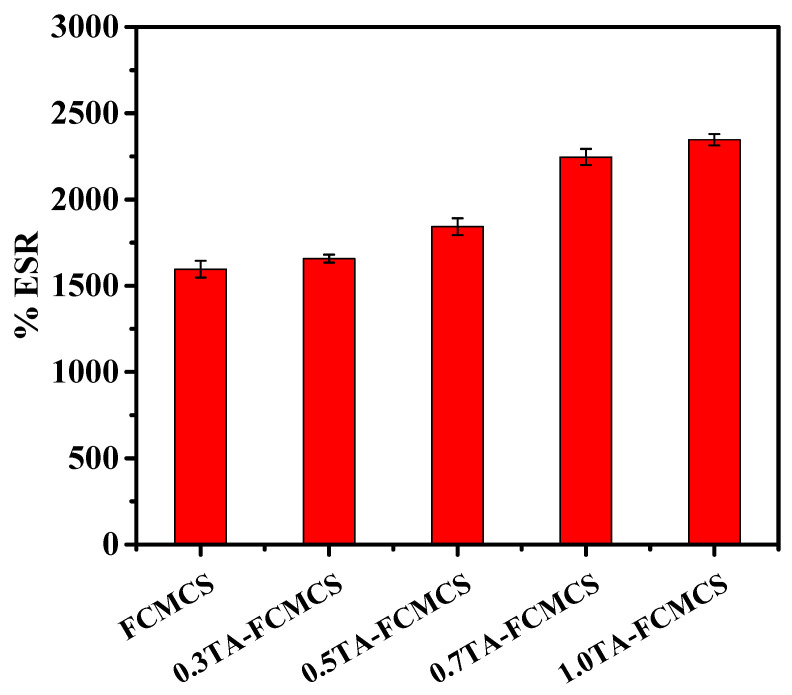
% equilibrium-swelling ratio (%ESR) of hydrogels.

Figure 3 displays the equilibrium-swelling properties of the FCMCS and TA-FCMCS hydrogels. The FCMCS hydrogel exhibited good swelling capacity (1595.92 ± 48.54%) under physiological conditions (pH 7.4). In comparison to the FCMCS hydrogel, 1.0TA-FCMCS had the highest swelling capacity (2346.32 ± 32.65%) under similar conditions, which could be attributed to the presence of TA in the hydrogel. The %ESR increased significantly with an increase in the TA content in the hydrogel networks. The greater water retention in the TA-FCMCS hydrogel’s architecture is due to the presence of hydrophilic groups of hydroxyl-rich TA and hydrophilic groups of FCMCS and PAM networks (–OH, –CO-NH_2_, and –COOH). Therefore, this hydrogel is suitable for wound-dressing applications.

### 2.3. Cytocompatibility Analysis

**Figure 4:**
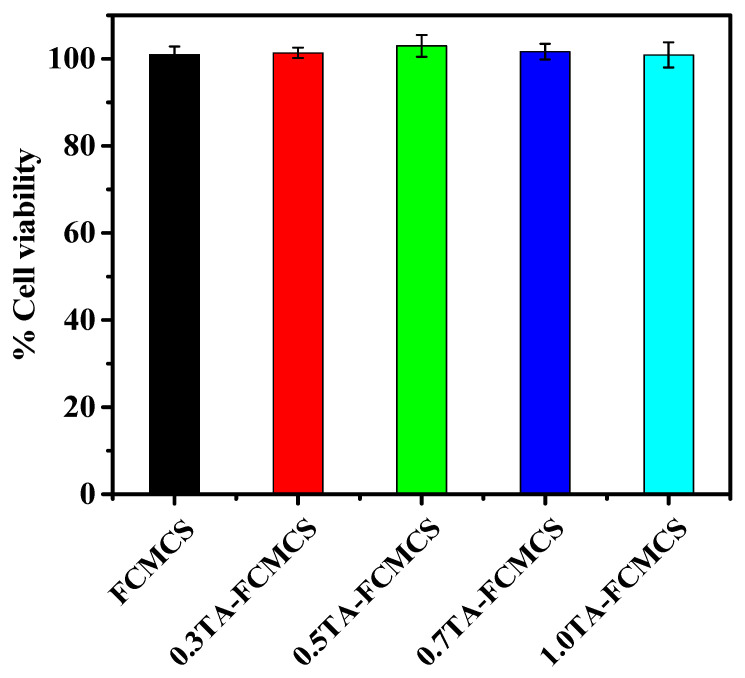
Cell viability of skin fibroblasts cells on hydrogels (determined using Prestoblue assay) after 72 h of incubation.

**Figure 5:**
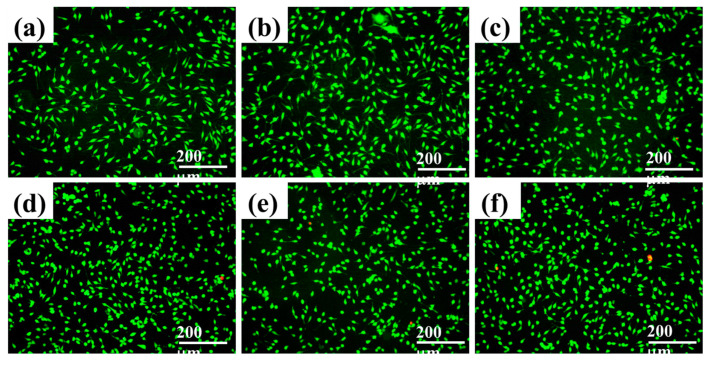
Live/dead images of skin fibroblasts cultured on (**a**) control, (**b**) FCMCS, (**c**) 0.3TA-FCMCS, (**d**) 0.5TA-FCMCS, (**e**) 0.7TA-FCMCS, and (**f**) 1.0TA-FCMCS hydrogels after 72 h of incubation.

Skin fibroblasts are model cells for wound healing because they play a critical role in the process [26]. Fibroblasts are responsible for producing ECM components such as collagen, elastin, and fibronectin, which form the structural framework of tissues. They are also involved in wound contraction, a process that reduces the size of a wound and promotes healing. Additionally, fibroblasts secrete growth factors and cytokines that promote cell proliferation and migration, which are essential for tissue repair. As a result, skin fibroblasts are widely used in in vitro wound healing models to study the mechanisms of wound healing and evaluate the efficacy of therapeutic interventions. Therefore, we used skin fibroblast cells as model cells to assess the cytocompatibility of the hydrogels. The cytocompatibility of the hydrogels was evaluated using the Prestoblue assay, and the outcomes are shown in Figure 4. The FCMCS and TA-FCMCS hydrogels containing different amounts of TA exhibited 100% cell activity, indicating their biocompatibility. FCMCS and TA are biocompatible materials that promote cell proliferation and migration, which may explain their excellent cell activity. The live/dead staining outcomes were consistent with the quantitative results (Figure 5), demonstrating that the majority of the cells on both the FCMCS and TA-FCMCS hydrogels displayed a normal, spindle-like morphology after a 72 h incubation period, with only a few dead cells (similar to the control). The results suggest that the TA-FCMCS hydrogels are a promising material for wound dressing, showing excellent cytocompatibility.

### 2.4. Antibacterial Activity

**Figure 6:**
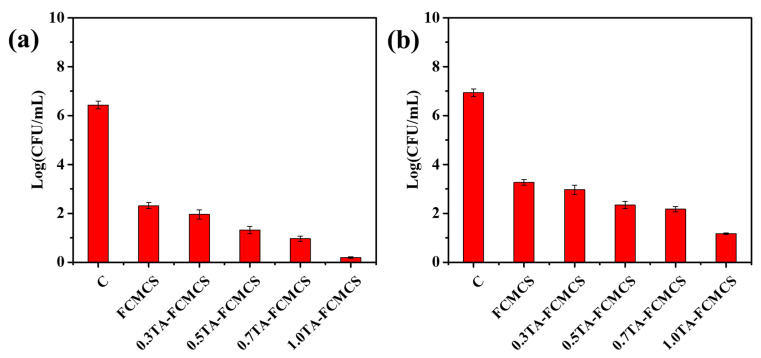
Antibacterial activity of hydrogels against (**a**) *Escherichia coli* and (**b**) *Staphylococcus aureus*.

TA has been shown to exhibit antibacterial activity against both Gram-positive and Gram-negative bacteria. Some studies have found that TA is more effective against Gram-positive bacteria, while others have found it to be more effective against Gram-negative bacteria. However, the exact effectiveness of TA against specific bacterial strains can vary depending on factors such as concentration, exposure time, and the method of testing. Recent studies have proven that FCMCS exhibits good antibacterial activity [27,28,29,30]. Thus, the antibacterial activities of the FCMCS and TA-FCMCS hydrogels were evaluated using the CFU method. Figure 6 illustrates that the growth inhibition values of the FCMCS hydrogels toward *Escherichia coli* and *Staphylococcus aureus* were about 65% and 52%, respectively, indicating that even FCMCS alone possesses an antibacterial effect. The antibacterial rates of the TA-FCMCS hydrogels against *Escherichia coli* (96%) and *Staphylococcus aureus* (89%) were significantly higher than those of FCMCS. Moreover, increasing the amount of TA improved the antibacterial properties of the TA-FCMCS hydrogels, indicating that the observed antibacterial performance was mainly due to both FCMCS and TA. Our findings suggest that the addition of TA to hydrogels can enhance their capacity to kill Gram-positive and Gram-negative bacteria [27,28,29,30]. The viability of the bacteria (*Escherichia coli* and *Staphylococcus aureus*) exposed to TA-FCMCS hydrogel was substantially reduced compared to that of the FCMCS hydrogel, indicating the critical role of TA in improving bactericidal properties. TA has been shown to have different mechanisms of action against these different types of bacteria. Gram-positive bacteria have a thick peptidoglycan layer in their cell walls, while Gram-negative bacteria have a thinner peptidoglycan layer and an outer membrane composed of lipopolysaccharides (LPSs). In Gram-positive bacteria, TA can disrupt the cell wall, leading to lysis and cell death. In Gram-negative bacteria, TA can disrupt the outer membrane and cause leakage of the intracellular contents, leading to bacterial death [27,28,29,30]. Overall, the incorporation of TA and FCMCS in the hydrogels is a promising approach for wound dressing, potentially improving wound-healing outcomes and reducing the risk of infection and inflammation. However, further research is needed to optimize the formulation and assess the safety and efficacy of these hydrogels in clinical settings.

### 2.5. Antioxidant Activity

**Figure 7:**
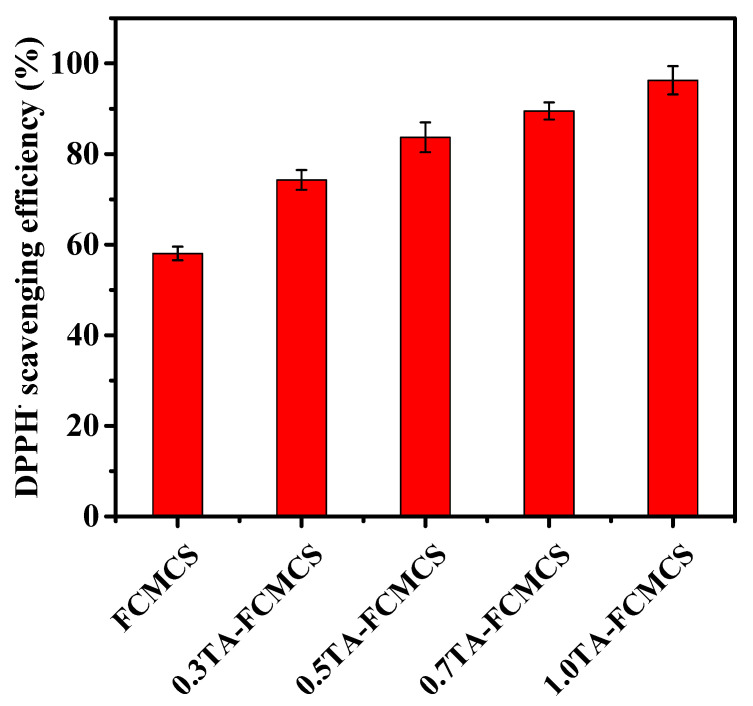
Antioxidant activity of hydrogels.

In general, CMCS shows good antioxidant properties due to the availability of carboxymethyl groups on its structure. In this study, the FCMCS hydrogel also shows good antioxidant properties because of the existence of the FCMCS polymer in the hydrogel system. FCMCS can act as an electron donor, which can help to neutralize free radicals and prevent oxidative damage. Additionally, CMCS has been found to increase the activity of antioxidant enzymes such as superoxide dismutase (SOD) and catalase, which can further protect cells from oxidative stress [31]. Furthermore, the incorporation of TA into the FCMCS hydrogel enhanced its antioxidant properties. To determine free-radical-scavenging activity, we used DPPH as a radical and found that the incorporation of TA into the FCMCS hydrogels significantly improved the radical-scavenging activity of the final product (Figure 7). Additionally, the clearance ratio of the radical of the TA-FCMCS hydrogels increased with the increase in TA content. The abundant hydroxyl groups in TA, which include catechol and pyrogallol, contribute to the high radical-scavenging activity of the TA-FCMCS hydrogels (96.2% for 1.0TA-FCMCS). Previous studies have reported that the antioxidant activity of polyphenols is associated with the hydroxyl groups in their structures [32]. Therefore, the radical-scavenging activity of the TA-FCMCS hydrogels is mainly attributed to the abundant hydroxyl groups in TA.

### 2.6. Tissue-Adhesive Properties

**Figure 8:**
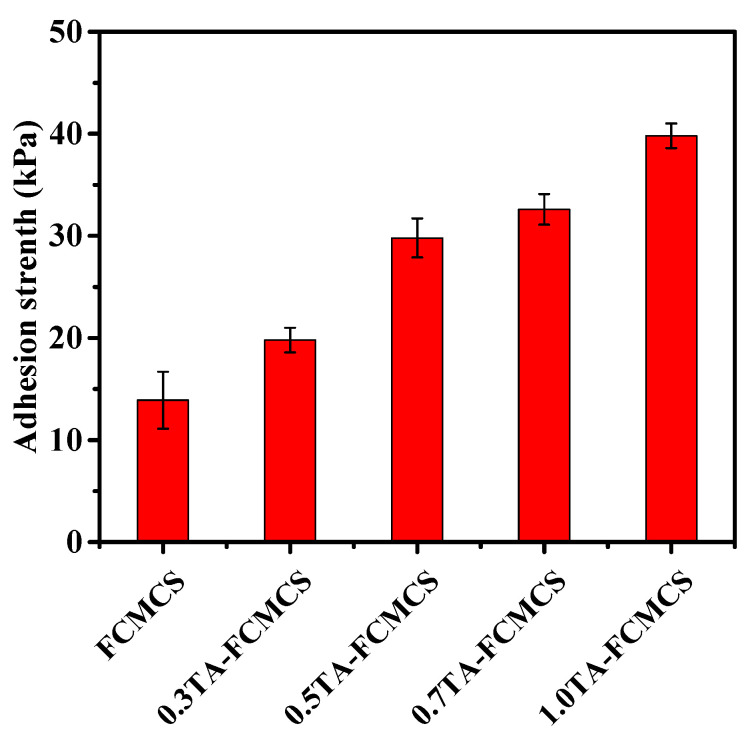
Adhesion strength of hydrogels to porcine skin.

As wound repair materials, the ability to adhere to tissues is a crucial characteristic of hydrogels, and various hydrogel adhesives designed for wound closure have been documented [33]. To evaluate the tissue adhesion ability of the TA-FCMCS hydrogels, tensile tests were performed on porcine skin, which functioned as a model tissue (Figure 8). The FCMCS hydrogel exhibited an average adhesive strength of 13.9 ± 2.8 kPa. In contrast, the amount of TA incorporated into the FCMCS hydrogels was significantly higher than that of the FCMCS hydrogels. The adhesion strength values of the 0.3TA-FCMCS, 0.5TA-FCMCS, 0.7TA-FCMCS, and 1.0TA-FCMCS hydrogels were 19.8 ± 1.2, 29.8 ± 1.9, 32.6 ± 1.5, and 39.8 ± 1.2 kPa respectively. The results confirmed that the adhesion strength of the TA-FCMCS hydrogel significantly improved when increasing the concentration of TA. This improvement could be attributed to the increased presence of phenol groups in the hydrogel network, which facilitated dynamic Schiff-base-binding reactions with amino groups in the skin tissue, thus enhancing interfacial adhesion. These findings suggest that the TA-FCMCS hydrogels exhibit diverse interfacial adhesion properties, indicating their potential for use as reliable medical adhesives for wound dressing.

## 3. Conclusions

We developed a multifunctional hydrogel using the free-radical polymerization method with TA and FCMCS incorporated in a PAM network. The hydrogels demonstrated a highly interconnected porous structure with nanoporous features and good swelling capacity. The antibacterial capacities of the 1.0TA-FCMCS hydrogels against *Escherichia coli* (96%) and *Staphylococcus aureus* (89%) were significantly higher than those of the FCMCS hydrogel. Additionally, the hydrogels exhibited antioxidant activity, particularly 1.0TA-FCMCS (96.2%). The FCMCS and TA-FCMCS hydrogels, containing different amounts of TA, exhibited 100% cell activity, indicating their biocompatibility, and demonstrated tissue-adhesive properties, with 1.0TA-FCMCS presenting an adhesion strength of 39.8 ± 1.2 kPa with respect to porcine skin. Overall, the physicochemical and biological properties of the hydrogels were greatly influenced by the concentration of TA. Based on its multifunctionality, the developed hydrogel has the potential to be used as a wound dressing for infected wounds. To produce better wound dressings, in vivo studies must be undertaken in future work.

## 4. Materials and Methods

### 4.1. Materials

The FCMCS (degree of deacetylation ≥80%) used in this study was obtained from the Endovision Company, Daegu, Republic of Korea. It was derived from Agaricus Bisporous Mushroom, with a molecular weight ranging from 200 KDa to 2000 KDa, a polydispersity of 7.1, and a viscosity between 20 and 1000 cps. Its deacetylation level was in the range of 80–98%. TA, APS, BIS, and N,N,N′,N′-Tetramethylethylenediamine (TEMED) were procured from Sigma-Aldrich, while AM was purchased from Dae-Jung chemical metal Co., Ltd., Gyeonggi-Do, Republic of Korea.

### 4.2. Preparation of TA-FCMCS Hydrogels

To prepare the multifunctional hydrogels, 2 wt% FCMCS solution was prepared in DDW. A total of 12.5 mL of FCMCS solution was mixed with 2.0 g of AM and stirred until it reached a homogeneous solution. Next, we sequentially added BIS (5 mg), APS (50 mg), and TEMED (10 µL) to the reaction system and filled molds. After 30 min, the hydrogels were formed; then, they were immersed in water to remove unreacted monomers, crosslinker, and initiator. Finally, the resulting FCMCS hydrogels were separately soaked in 50 mL of TA solution (0.3, 0.5, 0.7, and 1.0 wt% prepared and maintained at pH 1.2 via adding 0.1 M HCl solution) at 60 °C for 12 h. Finally, the hydrogels were removed, repeatedly washed with DDW, and freeze-dried for further analysis. The hydrogel formulations were prepared with respect to varying TA concentrations, namely, 0, 0.3, 0.5, 0.7, and 1.0 wt%, and labeled FCMCS, 0.3TA-FCMCS, 0.5TA-FCMCS, 0.7TAFCMCS, and 1.0TAFCMCS, respectively. 

### 4.3. Characterization

The Fourier transform infrared spectra (FTIR) of FCMCS, TA, and TA-FCMCS hydrogels with different amounts of TA were obtained using a Perkin Elmer spectrometer, for which measurements were taken across a wavenumber range of 4000 to 500 cm^−1^. To prepare the TA-FCMCS hydrogel samples for imaging, they were freeze-dried and attached to a metal stage before being coated with a layer of platinum using a sputter coater. The microstructures of the hydrogels were then visualized using a Hitachi S-4800 scanning electron microscope (SEM), and the pore diameters were determined using ImageJ software. Specifically, the XRD patterns of TA-FCMCS hydrogels were recorded using a Bruker AXS D8 advance diffractometer in Bragg–Brentano geometry, which involves a sample mounted on a rotating stage and the use of an X-ray source. The X-ray radiation used was Cu Kα line radiation, which has a wavelength of approximately 1.54 A^o^. 

### 4.4. Equilibrium-Swelling Studies

To determine the percentages of the equilibrium-swelling ratios (%ESR) of the TA-FCMCS hydrogel samples, dried hydrogels were first weighed and then immersed in PBS at 37 °C for 72 h. After each immersion period, the TA-FCMCS hydrogels were removed, the excess surface liquid was gently blotted, and the weights of the swollen hydrogels were recorded. The %ESR was calculated using the following formula:

$$\%\mathrm{ESR}=\frac{\left(W_{s}-W_{i}\right)}{W_{i}}\times 100$$

where W_i_ represents the initial weight of the hydrogel and W_s_ represents the weight of the hydrogel after immersion in PBS for 72 h of incubation. Each group of samples was analyzed a minimum of three times to ensure accuracy and reproducibility. The results were presented as mean values with corresponding standard deviations.

### 4.5. Biocompatibility Analysis

Prestoblue assay was performed to evaluate the biocompatibility of hydrogels. To evaluate the biocompatibility of TA-FCMCS hydrogels, skin fibroblast cells (CCD-986sk) were utilized. The hydrogel samples were first sterilized in ethanol, washed with phosphate-buffered saline (PBS), and then fixed in 24-well plates with DMEM medium. In each well, 5 × 10^4^ cells were seeded and incubated for 72 h. After removing the media, Prestoblue solution (100 µL from a 1:10 dilution) was added and incubated for 2 h. The optical density (OD) of the samples was measured at 570 and 600 nm using a microplate reader to calculate cell viability. Viability values were calculated by normalizing the average OD treated hydrogels to the control groups. To further assess cell biocompatibility, cells were stained with 50 µL of calcein AM/ethidium homodimer-1 double-staining kit and incubated for 30 min at room temperature. The stained cells were visualized using an inverted fluorescence microscope (Nikon Eclipse Ti, Genova, Italy).

### 4.6. Antibacterial Activity

The antibacterial efficacy of the freeze-dried TA-FCMCS hydrogels was assessed by incubating 50 mg of sterilized hydrogels with 1 mL of *Staphylococcus aureus* or *Escherichia coli* suspensions (with a concentration of 1 × 10^6^ CFU/mL) in a 24-well plate. Following 24 h of incubation at 37 °C, the bacterial suspensions were diluted with PBS to a concentration of 100 CFU/mL. Subsequently, 100 μL of the diluted bacterial medium was spread on a plate count agar and incubated for another 24 h at 37 °C to enumerate bacterial colonies. Each sample group was analyzed at least three times, and the results were presented as the mean and standard deviation.

### 4.7. Antioxidant Activity

In order to evaluate the antioxidant activity of the TA-FCMCS hydrogels, the DPPH free-radical-scavenging assay was conducted. A 100 μM solution of DPPH was prepared by dissolving DPPH in ethanol. The hydrogel samples were ground into a paste and dispersed in ethanol to produce a hydrogel dispersion with a concentration of 100 mg/mL. Subsequently, equal volumes (2 mL) of the DPPH ethanol solution and the hydrogel dispersion were mixed in the dark and left to incubate for 1 h. The absorbance of the resulting mixture was measured at 517 nm using a UV–vis spectrophotometer. The percentage of DPPH scavenging was then calculated using the formula provided below:

$$\mathrm{DPPH}\;\mathrm{assay}=\frac{\left(\mathrm{Absorbance}\;\mathrm{of}\;\mathrm{control}-\mathrm{Absorbance}\;\mathrm{of}\;\mathrm{hydrogel}\right)}{\mathrm{Absorbance}\;\mathrm{of}\;\mathrm{control}}\times 100$$

Each group of samples was analyzed a minimum of three times to ensure accuracy and reproducibility. The results were presented as mean values with corresponding standard deviations.

### 4.8. Tissue-Adhesive Properties

To evaluate the adhesive performance of the TA-FCMCS hydrogels, they were applied onto porcine skin surfaces [34]. The adhesive strength of the hydrogels was evaluated using an adhesive strength test. Two pieces of porcine skin were then overlapped, and the hydrogel was applied onto the overlapping area (25 × 20 mm bonding area). Adhesive stress was measured using MCT 2150 tensile tester (A&D Co., Ltd., Tokyo, Japan) with a stretching speed of 10 mm/min. Each group of samples was analyzed a minimum of three times to ensure accuracy and reproducibility. The results were presented as mean values with corresponding standard deviations. 

## Data Availability

Not applicable.
